# Spontaneous ectopic head formation enables reversal of the body axis polarity in microscopic flatworms

**DOI:** 10.1098/rspb.2025.1941

**Published:** 2025-10-29

**Authors:** Katarzyna Tratkiewicz, Ludwik Gąsiorowski

**Affiliations:** ^1^ Institute of Evolutionary Biology, Faculty of Biology, University of Warsaw, Warsaw 02-089, Poland

**Keywords:** regeneration, paratomy, asexual reproduction, Platyhelminthes, Microturbellaria, Catenulida, *Stenostomum*

## Abstract

In most of the animals, the antero-posterior axis is specified during early embryogenesis. However, in the organisms that undergo somatic asexual reproduction, constant re-establishment of the body axis occurs during each asexual act in the context of the fully formed adult body. In microscopic flatworms from the genus *Stenostomum* the new head and tail structures are inserted in the pre-existing body plan during the asexual process known as paratomy. Here, we report a spontaneously occurring developmental error that results in the formation of worms with double heads at opposite ends of their bodies, lacking posterior pole identity. In the set of experiments, we show that the double-head phenotype is not heritable on the organismal level. Worms originating from the sectioning or fission of the double-head animals give rise to the healthy populations that do not display the erroneous asexual development. We also demonstrate that the piece of the worm with ectopic head can survive, regenerate the tail on its previously anterior pole and resume asexual reproduction. Effectively, such regeneration allows stable reversal of the body axis polarity without impairment of the survival or reproductive abilities of the animal, an exceptionally rare phenomenon among bilaterians.

## 1. Background

In most of the animals, the specification of the body axis occurs early on during embryogenesis and represents one of the crucial developmental events, during which polarity of the adult animal is established [1–3]. However, in the organisms that engage in the somatic asexual reproduction, body axes of new individuals have to be specified within the context of the fully formed adult body, either by establishment of the new axes or by developing new individuals along the already established polarity [4–14]. There are three basic types of somatic asexual reproduction that differ in regards to the alignment of the body axes of maternal organisms and their asexual progeny, as well as the sequence of the formation of new tissues during the asexual process. The most widespread type of asexual reproduction is budding, in which new individuals (zooids) bud off from the maternal organism with their body axes specified *de novo* and not aligned with the axis of the maternal animal [4–9,15–17]. In contrast to budding, the body axes of the maternal individual and the newly formed zooids are aligned and continuous in two other modes of somatic asexual reproduction: architomy and paratomy. The main difference between those types is that in case of architomy, the maternal organism first divides and then regenerates missing body parts, while in paratomy, first the new structures of the zooid are formed within the maternal organism, and only later do the progeny separate [17–19].

On the mechanistic level, important differences between paratomy and architomy also concern the molecular and developmental control of the process. Architomy can be mostly seen as a form of whole body regeneration that follows autotomous division of the animal [20], and both processes are evolutionarily tightly linked [21]. Consequently, the development of a missing body part (anterior or posterior) is primarily triggered by its absence and the antero-posterior (A-P) body axis of the progeny is re-established after the fission [20]. In contrast, during paratomy, the new anterior and posterior structures develop in the functional, unsevered body. Therefore, the specification of the body axes of newly developing zooids has to occur within context of the existing polarity and requires more precise orchestration, with a gradual duplication of the entire A-P patterning [20]. Concordantly, paratomy seems to rely on different molecular mechanisms from regeneration [11].

In flatworms (Platyhelminthes), asexual reproduction evolved many times independently, resulting in different developmental modes being used by particular groups [17,22,23]. Both paratomy and architomy are attested among platyhelminths, the former in groups Catenulida and Macrostomorpha and the latter in planarians [17,18,22]. Any somatic growth of flatworms depends on the pluripotent stem cells [24–29] that also take part in the process of asexual reproduction [14,25,30,31]. Under normal circumstances, differentiation of the flatworm stem cells is controlled by the A-P gradients of signalling molecules that precisely control the axial identity of the developing structures [32–36], even in those groups that do not engage in asexual reproduction [37,38]. Concordantly, the experimental disturbance of those signalling molecules can lead to the formation of ectopic head or tail tissues [34–36,39]. In planarians, which can reproduce asexually through architomy, the development of surplus heads during the regeneration process can also occur spontaneously [40–42] or as a result of extensive dissections [43,44].

In addition to planarians, the development of ectopic heads among platyhelminths has been reported so far only in catenulids [45,46], a group of microscopic free-living flatworms that reproduce asexually by paratomy and, similarly to planarians, are capable of full body regeneration [14,17,25,47–49]. In the series of experiments devoted to heritance and ageing in a catenulid *Stenostomum incaudatum*, Sonneborn observed that ageing worms (originating during paratomy from the most frontal zooid and therefore inheriting the ‘old’ head) tend to display erroneous development after a limited number of divisions [45]. These malformations included perturbations in specification of the A-P polarity that resulted in the formation of ectopic head and tail tissues, eventually leading to the death of the worm. Interestingly, similar phenotypes were also observed by the same author after worms were exposed to a toxic environmental agent, lead acetate [46].

Here, we report that development of ectopic heads can also happen spontaneously in another catenulid species, *Stenostomum brevipharyngium*, which is emerging as a new catenulid model [25,49,50]. Particularly, we observed animals in which ectopic heads developed instead of tail tissues during paratomy. This malformation produced anterior double-headed zooids with heads at both ends, followed by normal zooids with a regular head and tail. In series of experiments, we set out to test the viability of the fragments with the ectopic, posterior-facing heads, as well as their potential for regeneration and asexual reproduction. In contrast to the observations of Sonneborn, we show that the phenotype in *S. brevipharyngium* is not lethal and does not seem to be related to ageing of the animal. We also document that the zooids with a head developed on the posterior end can permanently reverse the polarity of their body axis. These results shed new light on the extreme developmental plasticity of *Stenostomum* that allows it to regulate even dramatic shifts in the organization of its body plan.

## 2. Methods

### (a) Animal husbandry

*S. brevipharyngium* cultures were ordered in 2010 from Connecticut Valley Biological Supply as *Stenostomum* sp. and since then maintained in the laboratory. The species identification followed the key to the North American species of *Stenostomum* [51]. The cultures were maintained in standardized Chalkley’s Medium (CM) at 20°C in the dark and fed ad libitum with the unicellular eukaryote *Chilomonas paramecium*. Under these conditions, the animals reproduce exclusively asexually and do not form gonads.

### (b) Animal fixation

For both antibody staining and RNA *in situ* hybridization, we used the same fixation procedure. The animals were first anaesthetized in 1.44% (w : v) MgCl_2_ in CM for *ca* 10 min. When the worms stopped active movements, they were fixed in 4% (v : v) formaldehyde in PBS + 0.1% Tween-20 detergent (PTw) for 30 min. Then the animals were washed three times in PTw and either stored at 4°C (for antibody staining) or washed in ultrapure deionized water, dehydrated in 100% methanol and stored in fresh 100% methanol in −20°C (for RNA *in situ* hybridization).

### (c) Antibody staining

The animals were washed three times in PBS + 0.1% bovine serum albumine + 0.1% Triton X (PBT) for 15 min at room temperature (RT) and then incubated for 30 min in 5% normal goat serum dissolved in PBT (PBT + NGS), also at RT. Next, we incubated worms overnight (ON) at 4°C in primary antibodies dissolved in PBT + NGS. We used the following primary antibodies: mouse anti-tyrosinated tubulin, Sigma T9028 (dissolved at 1 : 500) and rabbit anti-serotonin (5HT), Sigma S5545 (dissolved at 1 : 250). On the next day, the worms were washed six times with PBT for 15 min and incubated for 30 min in PBT + NGS, both at RT. The animals were subsequently incubated ON at 4°C in secondary antibodies dissolved in PBT + NGS. We used the following secondary antibodies: goat anti-mouse, conjugated with Alexafluor488, Thermo Fisher A-11001; and goat anti-rabbit, conjugated with Alexafluor647, Thermo Fisher A-21244 (both at the concentration 1 : 250). After incubation in secondary antibodies all steps were performed at RT. The animals were washed three times in PBT, twice in PTw and incubated for 40 min in Hoechst 33 342 dissolved in PTw (1 : 5000). Then we rinsed the animals twice in PBT, and incubated for 1 h in Phalloidin, conjugated with Alexafluor555, Thermo Fisher A34055 (10 U ml^–1^ dissolved in PBT). Finally, the animals were washed in PBS, mounted in FluoromountG (Thermo Fischer, 00-4958-02) and left overnight at 4°C for tissue clearance and hardening of the mounting medium. The mounted specimens were investigated with the Olympus IX83 microscope with a spinning disc Yokogawa CSUW1-T2S scan head (normal asexual worms) or with the Nikon A1R MP confocal laser scanning microscope (double-head worms).

### (d) RNA *in situ* hybridization

For fluorescent RNA *in situ* hybridization chain reaction (HCR) v3.0 [52], we used the same DNA probe oligo pools as in [25] ordered at the Integrated DNA Technologies Germany GmbH (München, Germany). For the *in situ* staining, we used the same protocol as in [53]. The worms were rehydrated in Me-OH/PTw series at RT, washed four times in PTw and then prehybridized in hybridization buffer for 40 min at 37°C. Next, the animals were placed in HCR probe mixtures at 1 µM concentration in hybridization buffer and incubated ON at 37°C. On the next day, the probes were removed with four washes of probe wash solution (each 10 min at 37°C), followed by three washes in 5 × saline-sodium citrate buffer (SSC) + 0.1% Tween-20 at RT. Then, the worms were incubated in amplification buffer for 30 min at RT. In the meantime, the HCR hairpins (Molecular Instruments: B1H1-546, B1H2-546, B3H1-647, B3H2-647) were prepared by heating for 1 min 30 s at 95°C and then cooling at RT in the dark for 30 min. The hairpins were mixed in amplification buffer at 40 nM concentration and added to the samples. The animals were incubated in the amplification buffer with hairpins ON at RT in the darkness. On the next day, the samples were rinsed three times with 5 × SSC + 0.1% Tween-20, twice with PTw and incubated for 40 min in Hoechst 33 342 in PTw (1 : 5000). The stained specimens were washed once in PBS, mounted in Fluoromount G (Thermo Fischer, 00-4958-02), left ON at 4°C and then imaged with the Nikon A1R MP confocal laser scanning microscope.

### (e) Regeneration experiments

For the regeneration experiment the worms were anaesthetized with 1% (w : v) MgCl_2_ in CM for *ca* 10 min. The worms were cut with an eyelash under the dissecting scope and immediately transferred to a fresh CM. The regenerates were washed a few times with CM to remove the anaesthetic medium. Single regenerates were then put into 400 μl of CM and kept in darkness at 20°C with addition of 100 μl of concentrated *C. paramecium* culture (if not stated otherwise). The live regenerating worms were briefly anaesthetized with 1% (w : v) MgCl_2_ in CM, imaged on Keyence VHX-7000 Digital Microscope and then replaced into fresh CM.

### (f) Quantification and statistical analyses

For quantification of the regeneration success, we first assigned each worm to one of five categories that linearly described the severity of the observed outcome: dead (0), dying (1), retarded regeneration (2), regular non-reproductive (3) or regular asexual (4). Next, we used the two-sided Mann–Whitney U test in Python v. 3.9.12 to probe for the difference in distribution of the outcomes between control and experimental animals. To statistically test for differences in asexual reproduction between experimental groups, we calculated the slopes of the reproduction curves for single individuals in each condition using slope function in Microsoft Excel v. 16.77.1 (electronic supplementary material, table S1) and compared them with the slopes of the reproduction curves of the control worms using the two-sided Mann–Whitney U test in Python v. 3.9.12. U statistics and *p*-values for all comparisons are available in electronic supplementary material, table S2. For both analyses, we defined significance at the level of *p*‐value <0.05.

## 3. Results and discussion

### (a) Characterization and occurrence of the double-head phenotype

**Figure 1.**
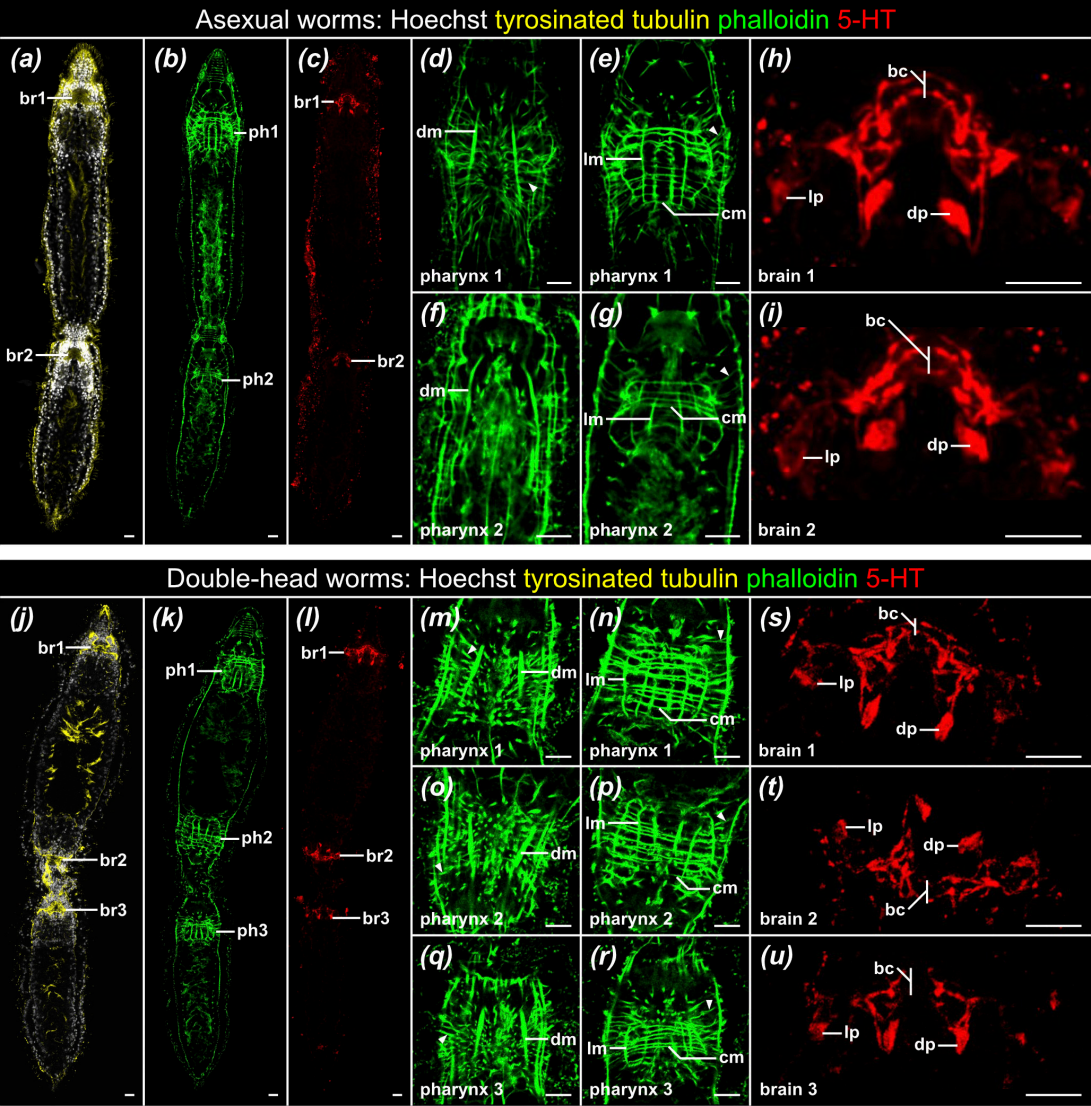
Morphological comparison of the wild-type asexual and double-head *Stenostomum brevipharyngium*. (*a–c*) General morphology of in the worm at the advanced stage of paratomy. (*d*–*g*) Detailed morphology of the pharynges in the asexual worm, showing the dorsal pharyngeal region (*d, f*) and the pharyngeal bulb (*e, g*). (*h, i*) Detailed morphology of the serotoninergic brain in the asexual worm. (*j*–*l*) General morphology of the double-head worm. (*m*–*r*) Detailed morphology of the pharynges in double-head worm, showing the dorsal pharyngeal region (*m, o, q*) and the pharyngeal bulb (*n, p, r*). (*s–u*) Detailed morphology of the serotoninergic brain in the double-head worm. Abbreviations: bc, brain serotoninergic commissure; br1*–*br3*,* brains 1−3; cm, circular pharyngeal muscles; dm, dorsal pharyngeal muscles; dp, dorsal serotoninergic perikaryon; lm, longitudinal pharyngeal muscles; lp, lateral serotoninergic perikaryon; ph1*–*ph3, pharynges 1−3. White arrowheads (*d*, *e*, *g*, *m–r*) indicate radial pharyngeal dilator muscles. Scale bars indicate 10 μm.

*S. brevipharyngium* is a microscopic flatworm that can be easily reared in the laboratory [25,49]. Under favourable conditions *S. brevipharyngium*, similarly to the other species of the genus*,* engages in asexual reproduction by means of paratomy [14,25,45,47,49,54]. During the process, which altogether takes roughly 4 days, the animals grow in size and, after reaching a certain length, they start to produce new anterior structures in the middle of their trunks, in the area referred to as the fission zone. First, the pluripotent stem cells start to divide and accumulate at the lateral sides of the gut, and later they differentiate into new head and pharyngeal tissues [30,49,54]. At the late stage of paratomy, the anterior structures of the new individual are fully formed in the middle of the A-P axis, giving rise to a chain that is composed of two zooids that are aligned along A-P axis and attached head-to-tail at the fission zone (figure 1*a*–*c*). In this advanced stage, the new, second zooid is equipped with pharyngeal and brain tissues that already resemble those of its maternal individual, both in terms of morphology and polarity (figure 1*d–i*).

In one of our routinely run laboratory cultures of *S. brevipharyngium,* we started to observe a bizarre developmental malformation that we nicknamed ‘double-head worms’. These animals look similar to the individuals in advanced stage of paratomy, but in addition to the anterior-facing head of the second zooid, they also have another, posterior-facing head, present at the posterior pole of the first zooid, just in front of the fission zone (figure 1*j–l*). When we inspected details of the middle heads, we could observe that all the structures in their pharynges and brains have reversed polarity when compared with the anterior and posterior heads in the chain (figure 1*m*–*u*); however, they did not show any obvious malformations of the head structures. For instance, we could detect the well-developed, yet mirrored, pharyngeal musculature, including circular, longitudinal, dorsal and radial muscles (figure 1*o,p*). Similarly, the main serotoninergic structures (dorsal and lateral perikarya, brain commissure) were present, albeit rotated by 180^o^ (figure 1*t*). This apparently normal organization of the ectopic head tissues suggests that these heads might be fully functional.

The double-head worms occurred spontaneously only in one of our cultures and were never very numerous—the Petri dish with hundreds of normal worms would yield fewer than 20 individuals, and often just a few at one time. The culture in which we observed the malformed animals was maintained according to the standardized protocol, with the same culturing medium, feeding and cleaning procedures as the other cultures in our laboratory. After discovering the double-headed worms, we screened the remaining cultures (*ca* 30 Petri dishes) but did not detect any additional individuals with this phenotype, suggesting that it is restricted to a single culture. After using most of the double-head worms for the experiments reported below, we propagated this culture into new dishes but the double-head worms were becoming decreasingly frequent and eventually they disappeared from the cultures. The entire outbreak of the double-head worms lasted altogether for *ca* 2 months.

In the meantime, we set out to investigate whether the surplus heads of the affected worms develop instead of the tail during paratomy, or whether they originate through the duplication of the entire A-P axis of the frontal zooids. To distinguish between those two scenarios, we needed to know whether the anterior zooid has any tail tissue located in the middle of its body (which would hint at the duplication of the A-P axis), or whether the tail identity is absent and replaced by the head tissue instead. In contrast to the head, which can readily be distinguished based on the morphology, the tail of *Stenostomum* is an elusive body region that does not harbour many distinct structures. However, a recently published single-cell atlas of *S. brevipharyngium* reported specialized hindgut cells located in the most posterior section of the gut that could be used as markers of the posterior identity, at least within the digestive system [25]. Therefore, to test for the relation of head and tail structures in asexual and double-head worms, we studied the expression of the brain marker, *MEF2C*, and hindgut marker, *SLC26A2*, in the animals at advanced paratomy and with the double-head phenotype.

**Figure 2.**
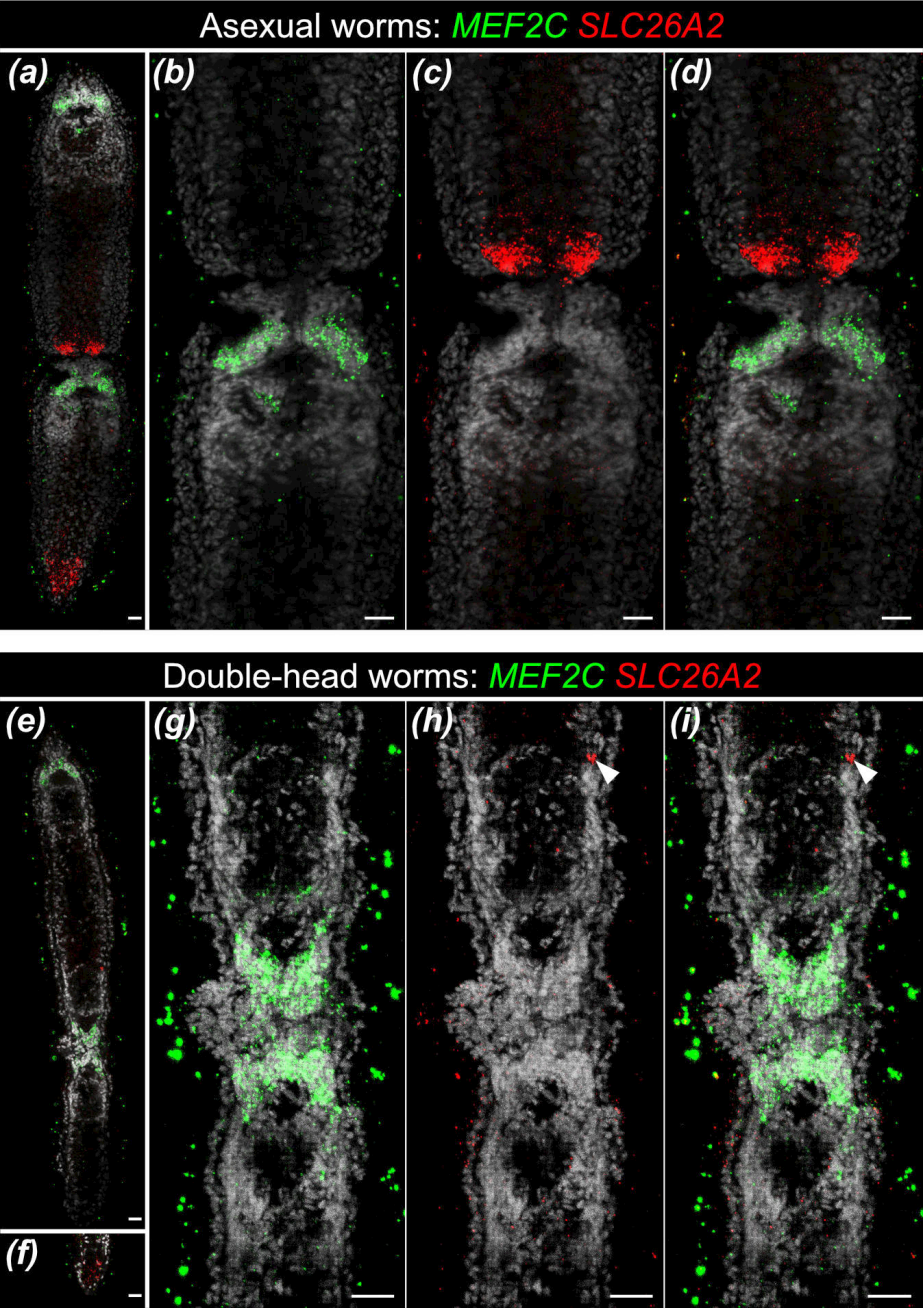
Comparison of the brain and hindgut markers’ expression between wild type asexual and double-head *Stenostomum brevipharyngium*. (*a*) Gene expression in the worm at the advanced stage of paratomy. (*b–d*) Detailed gene expression in the fission zone of the asexual worm. (*e*) Gene expression of in the double-head worm. (*f*) Gene expression in the posterior part of the posterior zooid. (*g–i*) Detailed gene expression in the fission zone of the double-head worm. White arrowheads indicate ectopic *SLC26A2*^+^ cell located in the pharyngeal region. Scale bars indicate 10 μm.

In the normal asexual worms, both heads express *MEF2C* and both tails express *SLC26A2* (figure 2*a*), and expression of both markers is strongly limited to their respective territories. In the fission zone, the *SLC26A2^+^* cells of the newly forming tail of the anterior zooid are adjacent to the *MEF2C^+^* brain cells of the second zooid (figure 2*b*–*d*). In the double-head worms, both anterior and posterior brains of the first zooid, as well as the brain of the second zooid are expressing *MEF2C* (figure 2*e*), while *SLC26A2* is expressed in the posterior part of the second zooid (figure 2*f*). In the fission zone, two clusters of the *MEF2C^+^* brain cells are located next to each other and the *SLC26A2^+^* cells are missing (figure 2*g*–*i*). The tail marker is generally absent from the anterior zooid (figure 2*e*), although some ectopic *SLC26A2^+^* cells can be present next to the pharynx of the posterior-facing middle head (arrowheads, figure 2*h*–*i*). Generally, these expression patterns indicate that the double-head worms emerge through the development of a new posterior-facing head, instead of the tail, during paratomy. Consequently, the anterior zooid of the double-head worms is altogether devoid of the tail identity.

### (b) Double heads are not heritable on the organismal level

Is the double-head phenotype related to some mutation, or is it a pathological condition that depends on some non-heritable factors? To address this issue, we performed set of experiments in which we assessed the heritability of the double-head phenotype.

**Figure 3.**
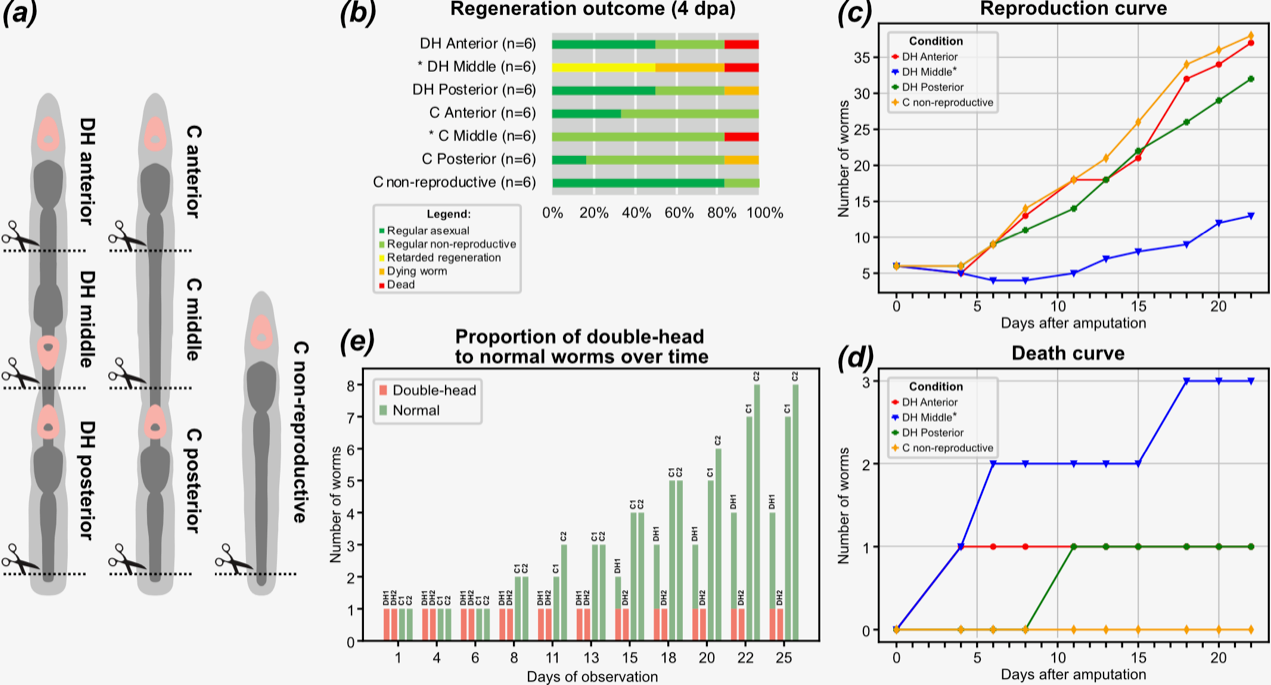
Experiments on the regeneration competence of different sections of the double-head worms and heredity of the double-head phenotype. (*a*) Schematic drawing of the experimental amputations. (*b*) Regeneration success at 4 days post-amputation (dpa) among experimental groups. (*c*) Reproduction curves of experimental groups. (*d*) Death curves of experimental groups. (*e*) Change in the proportions of double-head to normal worms among the descendants of two double-head (DH1 and DH2) and two control (C1 and C2) worms. Asterisks indicate significant differences from controls (*p*‐value < 0.05) as inferred with the two-sided Mann–Whitney U test. The primary data for panels (*c*) and (*d*) are available in the electronic supplementary material, table S1.

In the first experimental setup, we cut six double-head worms into three sections (figure 3*a*). An anterior fragment (DH anterior) encompassed the most anterior head and half of the first trunk. The middle fragment (DH middle) contained the posterior-facing head and the posterior half of the first trunk. The posterior fragment (DH posterior) originated by separation of the most posterior zooid of the chain. As a control, we cut six normal worms in advanced paratomy, following the same paradigm (figure 3*a*), which resulted in an anterior control fragment (C anterior), composed of the head and first half of the zooid; middle control fragment (C middle), containing second half of the trunk and a tail; and a posterior control fragment (C posterior), encompassing the most posterior zooid. Additionally, as another control group (C non-reproductive) we also took six normal worms, without double-head phenotype, that were not engaged in paratomy. Since anterior and middle fragments of the double-head worms were missing posterior identities (see above), we also cut off the most posterior tail tips of the DH posterior, C posterior and C non-reproductive (figure 3*a*). To avoid biases related to differences in regenerate sizes, we tried to select animals of similar dimensions for all experiments. The double-head worms with advanced middle heads and the worms in advanced paratomy were all of similar size because paratomy is initiated only in the large individuals. For the control non-reproductive worms, we chose the largest individuals that were not yet engaged in paratomy in order to maintain a similar regenerate sizes when compared to the pieces derived from asexual worms. Consequently, the sizes of all regenerates were similar immediately after amputations in all experimental setups, although not controlled precisely.

First, we scored the status of regenerates at 4 days post-amputation (dpa), checking whether the animals survived and what was their regenerative and reproductive status (figure 3*b*). Among non-reproductive control animals, all worms showed regular morphology and five were already engaged in paratomy. There was also a high regeneration success rate among control worms derived from sectioning of individuals in paratomy (figure 3*b*). All of the anterior pieces (C anterior) regenerated, with two of them already engaging in reproduction. Single middle and posterior fragments of control worms (C middle, C posterior) failed at regeneration, but the remaining ones regenerated well. None of the descendants of the middle pieces was engaged in paratomy at this stage, leading to their significantly different regeneration score when compared with the non-reproductive controls (figure 3*b*). This is likely owing to the fact that, in contrast with the other conditions, these animals needed to fully regenerate head tissues—which takes around 4 days in *S. brevipharyngium* [25,49]—before they could initiate asexual reproduction.

For both anterior and posterior fragments of double-head worms (DH anterior, DH posterior) single pieces failed at regeneration, but the remaining were healthy, did not develop ectopic heads, and half of them already showed symptoms of normal asexual reproduction, generally exhibiting the same outcome as control animals (figure 3*b*). The regeneration success rate of the middle fragments of double-head worms (DH middle) significantly differed from the control non-reproductive individuals (figure 3*b*). Among these pieces, one was dead, two were in advanced degeneration (resorption of the head, shrinking of the body) and three exhibited retarded regeneration. The latter animals were smaller than the control worms and either had heads looking like an early regenerative stage (two individuals) or additionally had slight bumps on the body (a single individual), but they were all single-headed. Altogether, we did not observe formation of ectopic heads in any of the worms originating from double-head ancestors during regeneration.

Next, we observed the double-head animals that survived the regeneration over the course of following 18 days, up to 22 dpa, and compared their reproduction and viability to the non-reproductive control worms (electronic supplementary material, table S1). The descendants of anterior and posterior fragments of the double-head worms were dividing at the same rate as control animals (figure 3*c*) and showed low overall mortality rate (one animal in each group, figure 3*d*). Three middle fragments of double-head worms died (figure 3*d*) and the remaining three resumed asexual reproduction between days 8 and 15, when the animals from the other groups had already undrgone 2−3 divisions (electronic supplementary material, table S1). In consequence, the descendants of the middle fragments showed a significantly delayed reproduction rate when compared with the other experimental groups or controls (figure 3*c*). In total, we observed 32 divisions among the descendants of anterior pieces and control worms, 10 divisions among the descendants of middle fragments and 27 divisions among the descendants of the posterior ones (electronic supplementary material, table S1). Again, we did not observe any double-head animals in any of the progeny of the initial six double-head individuals or controls during these fissions.

In the above-described treatments, the double-head worms have been cut, which might influence transmission of the phenotype to the next generation. Therefore, we set up another experiment to observe the heritability of the double-head phenotype in the intact animals. We took two double-head and two control worms and cultured them for 25 days, scoring the number of double-head and normal individuals over time (figure 3*e*). While one of the double-heads remained arrested in its asexual reproduction, the other divided after 2 weeks of observation, giving rise to a double-head maternal worm and a new, normal individual that originated by paratomous fission of the most posterior zooid. This normal animal kept dividing and did not produce further double heads, echoing the results of the first experiment.

Altogether, these experiments showed that the double-head phenotype that we observed in S*. brevipharyngium* is not heritable on the organismal level, so it cannot be attributed to, e.g. interindividual differences in the genetic control of the asexual developmental programme. What other potential explanation could there be for the formation of ectopic heads?

A similar phenotype, with the animals producing a head instead of a tail during paratomy, was reported by Sonneborn in the 1930s in *S. incaudatum* [45,46]. In this species, the double-head worms resulted from the ageing of the head of the most anterior zooid [45], and represented only a small fraction of a broader spectrum of developmental malformations observed with high prevalence under those conditions (e.g. bloating, ectopic tails, accumulation of hyaline spherules). In the case of our animals, the double-head phenotype was the only malformation observed in the culture, and other phenomena reported by Sonneborn as symptoms of ageing were not present. Therefore, the mechanisms underlying double heads in *S. incaudatum* and *S. brevipharyngium* are probably of a different nature. Ageing is also an unlikely cause of the double heads that we observed, taking into account our finding that in our experiment the most anterior (the oldest) head pieces of the double-head worms resumed normal asexual reproduction and their progeny did not show any malformations, while in Sonneborn’s experiments, after their development of age-related symptoms, the most anterior worms of the chains accumulated malformations and were eventually destined to die [45]. Moreover, our laboratory cultures of *S. brevipharyngium* have been kept in captivity for more than 25 years [49] (since 2019 by the last author), and the double-head phenotype has not been observed thus far, in contrast to what would be expected from the physiologically occurring symptoms of ageing. Instead, they were only restricted to a single animal culture in a relatively short time period (2 months).

Alternatively, the double-head phenotype might also be an effect of stochastic perturbances of the A-P patterning molecular systems caused by some unknown environmental cue. Perturbances of the A-P axis, including but not limited to double-head worms, have been reported in *S. incaudatum* as a result of intoxication with lead acetate [46]. However, environmental toxicity is an unlikely reason for the phenomenon that we observed in our cultures. First of all, the double-head worms occurred in a Petri dish that was kept under the same conditions as the other cultures in our laboratory. We used the same medium, food and labware to maintain all of them; therefore, any chemical contamination should manifest in more than a single dish. Moreover, within the affected culture, only a small fraction of individuals displayed the phenotype, while most of the worms in the same dish performed unaltered paratomy. Yet we cannot fully exclude the possibility that the double-head phenotype might represent a response to some unidentified toxic agent to which only a small fraction of individuals were sensitive owing to interindividual differences, related either to somatic mutations or environmental stress.

Another possible explanation for the occurrence, prevalence and heredity of the double-head phenotype is that it is related to mutations at the level of the ever-dividing adult pluripotent stem cells. In flatworms, the morphogen gradients along A-P axis pattern stem cells’ differentiation into the identities specific to particular body regions [32–35,37,38]. Although the exact molecular mechanisms maintaining A-P identity in *Stenostomum* remain unknown, these animals also rely on adult pluripotent stem cells for regeneration and asexual reproduction [14,25,30] and they therefore likely maintain A-P morphogen gradients that instruct stem cell differentiation and are involved in patterning of new structures during paratomy. The double-head *Stenostomum* might carry chimeric populations of stem cells that include a stem cell line that shows a faulty response to those gradients. For instance, mutated stem cells might respond erroneously to the morphogens instructing the development of posterior structures and develop into anterior elements instead. In such a case, the double-head phenotype would only manifest when the stem cells originating from the mutated line are present in the fission zone and contribute to the formation of the posterior end of the anterior zooid during paratomy. Consequently, after the mutated stem cells differentiate into head structures, their mutation could not be passed to next generation, effectively eliminating the phenotype from the population.

Interestingly, even though the double-head worms are now known to develop in two different species of *Stenostomum*, their occurrence seems to be limited only to the laboratory strains as such malformed worms are not reported in the natural populations. This might be explained by two factors. First of all, the presence of ectopic heads with reversed polarity is highly debilitating, as affected worms experience conflicting directional signals during movement. Indeed, we observed that the double-head worms displayed problems with swimming when compared with the regular individuals. Consequently, in the natural environment, such individuals would be more prone to predation and likely disadvantaged in grazing compared with the normal worms. Additionally, the species in which the malformation has been observed reproduce exclusively asexually in laboratory conditions. For this reason, these laboratory strains have a higher chance of accumulating various mutations in their stem cells that would be eliminated from natural populations when the worms engage in the sexual phase.

### (c) Double heads allow stable reversal of the body axis polarity

**Figure 4.**
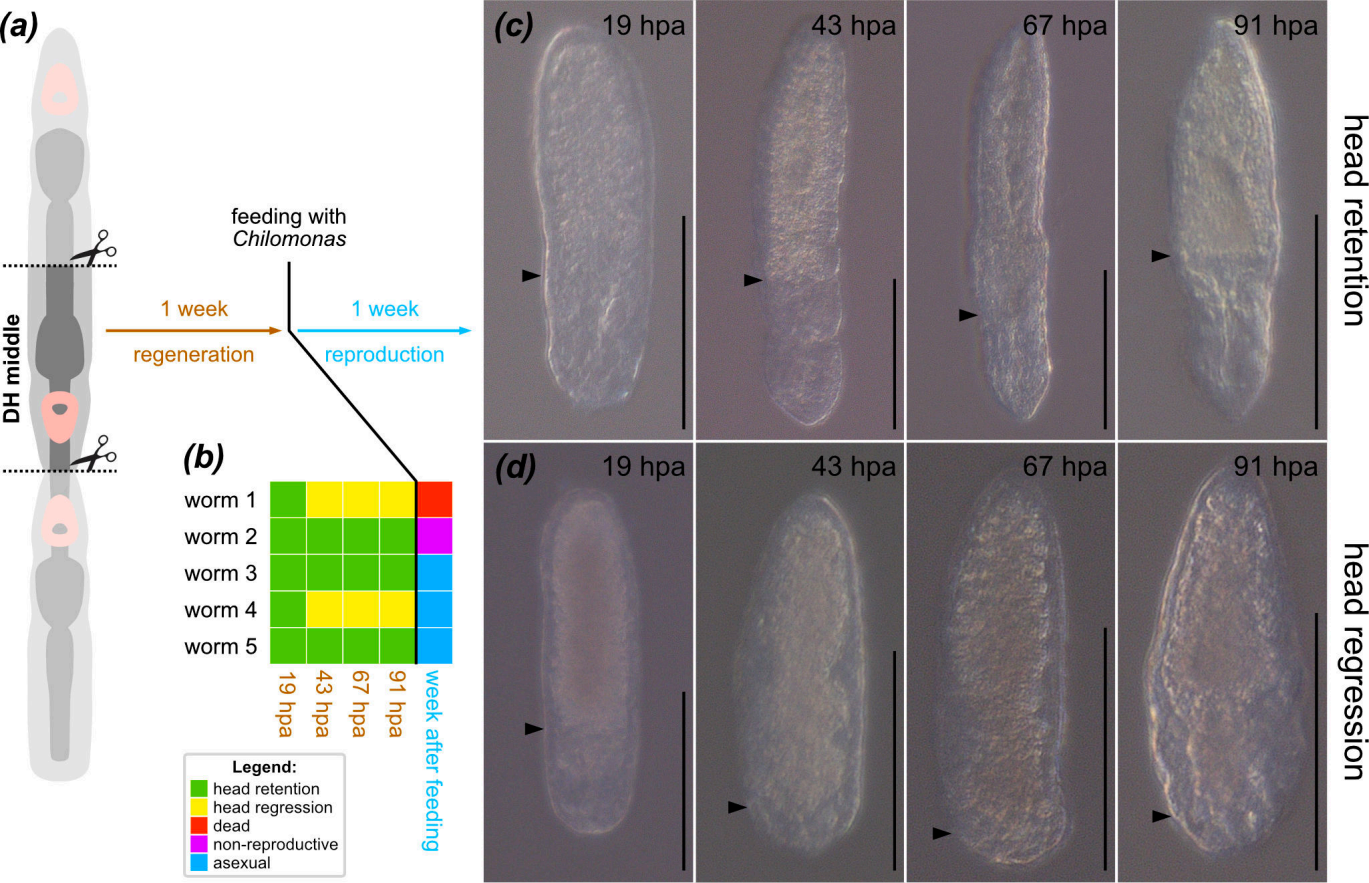
Reversal of the body axis polarity in the regenerating middle zooid of the double-head worms. (*a*) Experimental design. (*b*) Regenerative and reproductive status of the worms during the experiment. (*c*) Morphological time course of head retention in the middle zooid. (*d*) Morphological time course of head regression in the middle zooid. Black arrowheads indicate the most posterior extent of the gut tissue. Scale bars indicate 100 μm.

In our first experiment, we observed three animals with originally posterior-facing ectopic heads that survived bisection and established viable populations through asexual reproduction. Do those worms use the ectopic head as their new anterior pole or did they resorb it, regenerate the new one at the anterior pole and thus regulate their aberrant development? To investigate this matter, we conducted another experiment, this time focusing only on the middle fragment with a posterior-facing head and a shorter timescale (figure 4*a*). We cut five double-head worms in the same way as in the first experiment and kept the middle fragments in clean medium for 1 week to let them regenerate. Over this time, we observed every individual at 19, 43, 67 and 93 h post-amputation (hpa), which is the time required in *S. brevipharyngium* for complete regeneration [25,49]. On the 7th dpa the worms were fed and then observed for a week and scored for asexual reproduction. During the initial regeneration, three of the worms retained their posterior-facing heads (figure 4*b*,*c*) and did not grow new head structures until they engaged in paratomy, during which the new head structures formed normally at the fission zone, facing in same direction as the first head. Two worms retained their posterior-facing heads for *ca* 20 hpa but later began resorbing the ectopic head (figure 4*b*,*d*). One of those individuals died within 2 weeks post-amputation, while the other regenerated a head (although we could not determine at which end) and resumed normal asexual reproduction (figure 4*b*).

Notably, the animals that retained the ectopic head sustained a complete 180^o^ reversal of their A-P body axis, indicating that the reversal is stable on the organismal level and not only temporarily dependent on some external factors. As the descendants of the double-head animals do not show errors related to formation of the anterior or posterior structures and are capable of normal asexual development, it appears that their A-P patterning becomes fully regulated following the bisection and regeneration. While the brain and pharyngeal tissues of the middle fragment originated during paratomy with the reversed orientation, those pieces also retained trunk tissues originating from the ancestral un-inverted animal, including some that exhibit differentiation along the A-P axis—e.g. body-wall musculature, protonephridium or gut [25,55,56]. The fact that the worms were able to resume normal physiology, despite reversal of those vital organ systems in relation to their heads, points towards extreme physiological plasticity of their body plan. Such flexibility might be related either to relative simplicity of their organs or to the ability to dynamically remodel the tissues owing to the presence of the pluripotent stem cells [25,30].

## 4. Conclusions

We report here a rare double-head phenotype in *Stenostomum* flatworms that originates through the erroneous, yet spontaneous, formation of head tissues, instead of tail, during the asexual process of paratomy. While formation of the double heads in *Stenostomum* has previously been attributed to ageing [45] and environmental toxicity [46], our findings suggest that it may also result from other, as yet undetermined causes—possibly linked to chimerism in adult pluripotent stem cells or stochastic perturbances of the A-P patterning molecular systems.

A similar phenomenon (ectopic head formation, followed by regeneration of normal posterior structures on the opposite end) has been reported in some animals, e.g. cnidarians [6] or planarians [40–43]. In case of a planarian *Planaria maculata,* double-head formation and the following reversal of the body axis require two rounds of cutting and regeneration (including generation of very narrow pieces that hardly contain any tissues) [43]. In consequence, the individuals with the reversed polarity do not contain much tissue from the original, un-inverted worm. In the case of *Stenostomum*, the double heads occur spontaneously and contain large fragments of the regular animals that are inherited by the worms with reversed polarity, which is similar to what has been reported for another planarian, *Girardia dorotocephala* [40]. Similarly to catenulids, planarians are capable of whole-body regeneration and asexual reproduction, but they reproduce asexually through architomy, and not paratomy. Therefore, it seems that stable reversal of body axis polarity is rather related to the ability to regenerate the entire body and not to the particular mode of asexual reproduction.

We hypothesize that developmental plasticity, driven by the presence of adult pluripotent stem cells and continuous tissue renewal [24–26], allows flatworms to survive such a dramatic developmental event. The example of platyhelminths shows that whole body regeneration based on pluripotent stem cells provides a flexible developmental framework in which even a reversal of the A-P axis of the adult bilaterian body can be accommodated. This is related to the fact that whole body regeneration requires the ability to regulate large-scale rearrangements of the tissues and organ systems that are often inflicted by wounding. At the same time, the adult pluripotent stem cells allow unprecedented flexibility in reparation and remodelling of the remaining tissues. Whether such plasticity among bilaterians is only restricted to flatworms remains an open question, as reports on spontaneously occurring developmental malformations of this scale are rare. It is possible that flatworms are particularly prone to such developmental errors but at the same time able to regulate them owing to their unique system of tissue homeostasis based on the adult pluripotent stem cells controlled by embryonic-like morphogen gradients. This hypothesis could be rejected if similar double-headed individuals capable of regulating reversal of the A-P axis are reported from other groups of animals that regenerate heads without an adult pluripotent stem cell system, e.g. annelids [57].

*Stenostomum* has lately regained its interest among biologists studying regeneration, asexual reproduction and stem cell biology, as an emerging and intriguing model that differs in many respects from the other flatworms [14,25,48,49]. In recent years, an increasing number of molecular tools have been developed to study these peculiar worms [14,25,49,58], but our understanding of their basic biology still remains limited. The phenomenon that we report here is a prime example of how little we know about the developmental capacities of *Stenostomum*.

## Data Availability

The data analysed during this study are included in this article and its supplementary materials. The unprocessed microscopy files are deposited at Dryad and can be accessed online [59]. Supplementary material is available online [60].
